# Supplementary material for: A Novel Method for the Early Detection of Single Circulating, Metastatic and Self-Seeding Cancer Cells in Orthotopic Breast Cancer Mouse Models
Source: Cells. 2024 Jul 9;13(14):1166. doi: 10.3390/cells13141166 (PMC11275056; doi:10.3390/cells13141166)
Supplement: Supplementary file 1 [file cells-13-01166-s001.zip › cells-3054742-supplementary.pdf]

A Novel Method for the Early Detection of Single Circulating, Metastatic and Self-Seeding Cancer Cells in Orthotopic Breast Cancer Mouse Models

Muhammad Murad [1], Yanjiang Chen [1,2], Josephine Iaria [1,2], Adilson Fonseca Teixeira [1,2] and Hong-Jian Zhu [1,2,*]

[1] Department of Surgery, The Royal Melbourne Hospital, The University of Melbourne, 5th Floor Clinical Sciences Building, Parkville, VIC 3050, Australia; muhammadmura@student.unimelb.edu.au (M.M.); yanjiang.chen@usz.ch (Y.C.); jiaria@unimelb.edu.au (J.I.); afonsecateix@student.unimelb.edu.au (A.F.T.)

[2] Huagene Institute, Kecheng Science and Technology Park, Pukou District, Nanjing 211806, China

* Correspondence: hongjian@unimelb.edu.au; Tel.: +61-3-8344-3025; Fax: +61-3-9347-6488

SUPPLEMENTARY MATERIALS

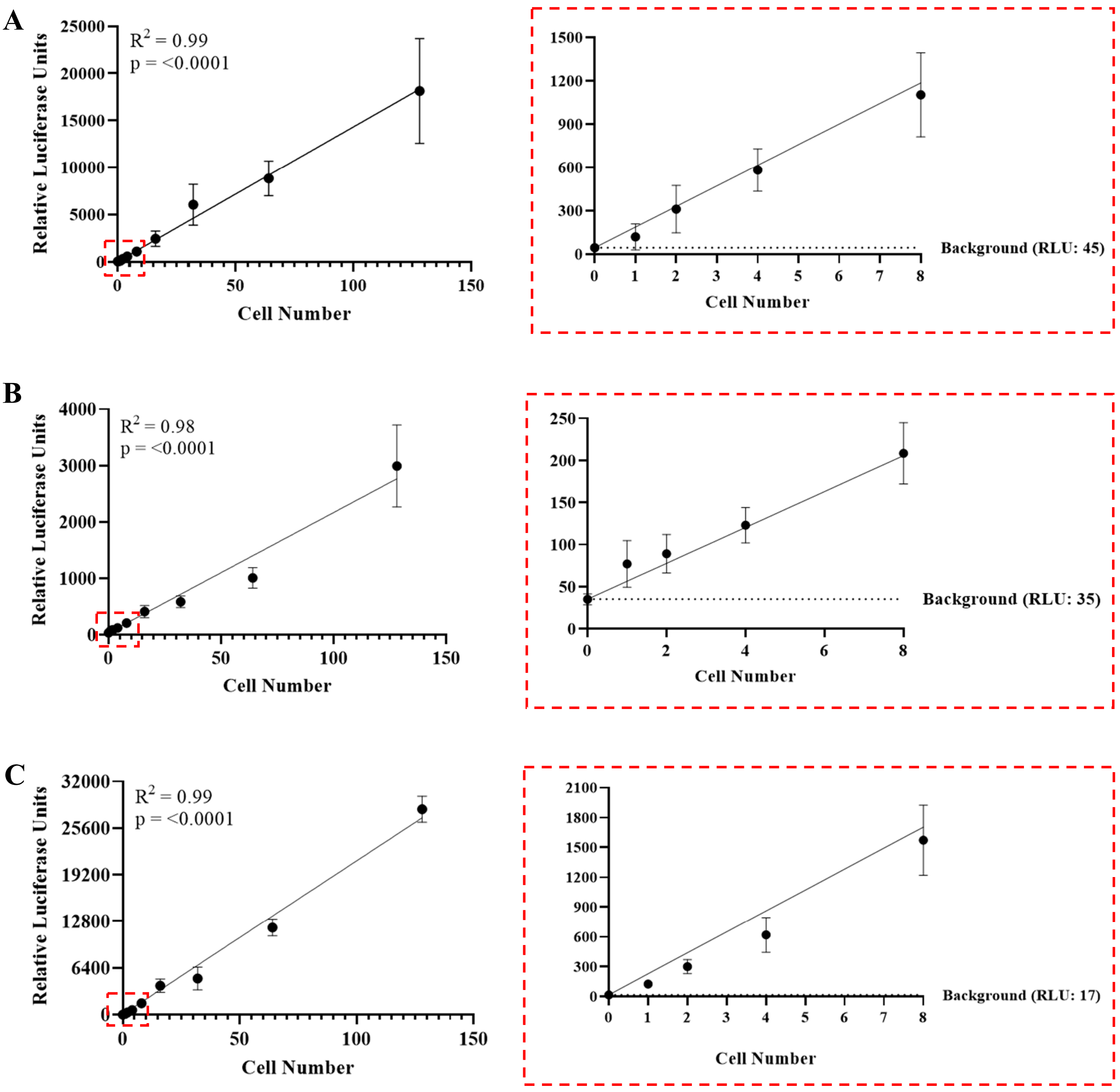

Figure S1: Luciferase-labelled tumour cells spiked in brain samples are detected at single-cell level. Detection and quantification of MDA.Gluc **(A),** MCF7.Gluc **(B),** and 4T1.Fluc **(C)** cells spiked in 5 mg of mouse brain samples. Left panels show results obtained with all dilution points analysed. Right panels show enlarged regions of left panels, highlighting results obtained with 0-8 cells. Data is represented as mean ± SEM and corresponds to relative luciferase units (RLU) obtained from six readings. Linear correlation was determined by best-fit linear regression analysis.

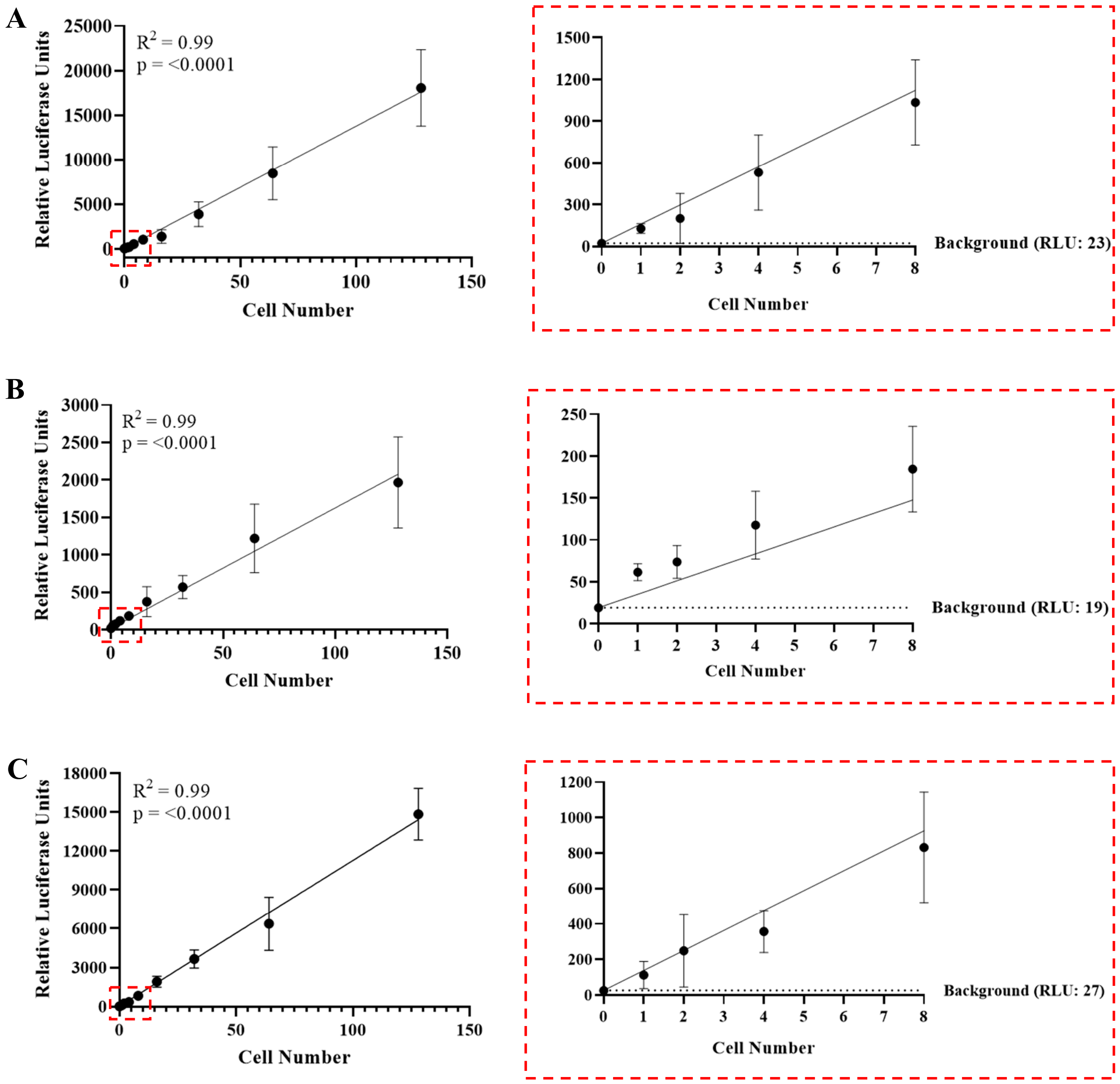

Figure S2: Luciferase-labelled tumour cells spiked in lung samples are detected at single-cell level. Detection and quantification of MDA.Gluc **(A),** MCF7.Gluc **(B),** and 4T1.Fluc **(C)** cells spiked in 5 mg of mouse lung samples. Left panels show results obtained with all dilution points analysed. Right panels show enlarged regions of left panels, highlighting results obtained with 0-8 cells. Data is represented as mean ± SEM and corresponds to relative luciferase units (RLU) obtained from six readings. Linear correlation was determined by best-fit linear regression analysis.

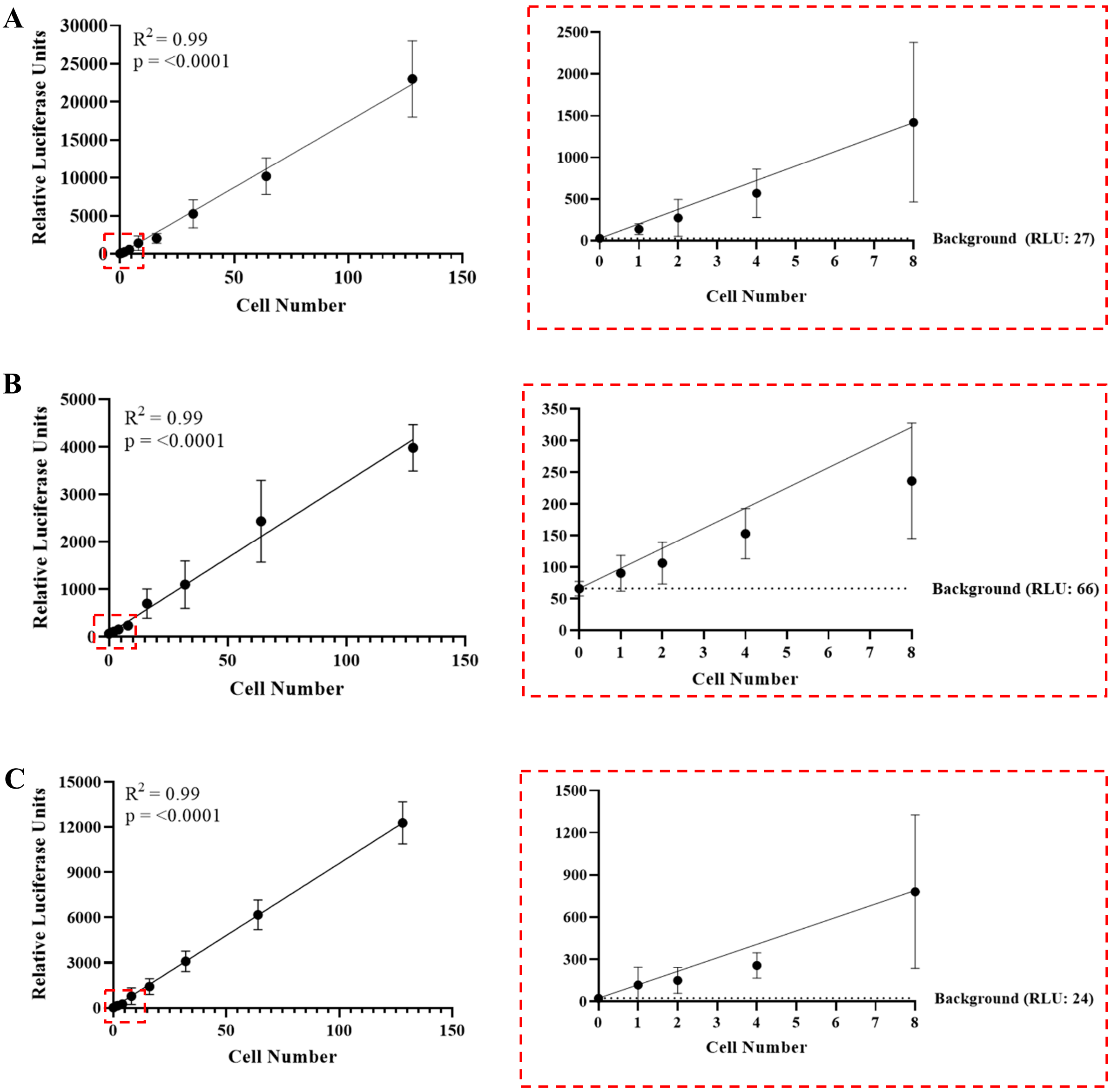

Figure S3: Luciferase-labelled tumour cells spiked in liver samples are detected at single-cell level. Detection and quantification of MDA.Gluc **(A),** MCF7.Gluc **(B),** and 4T1.Fluc **(C)** cells spiked in 5 mg of mouse liver samples. Left panels show results obtained with all dilution points analysed. Right panels show enlarged regions of left panels, highlighting results obtained with 0-8 cells. Data is represented as mean ± SEM and corresponds to relative luciferase units (RLU) obtained from six readings. Linear correlation was determined by best-fit linear regression analysis.

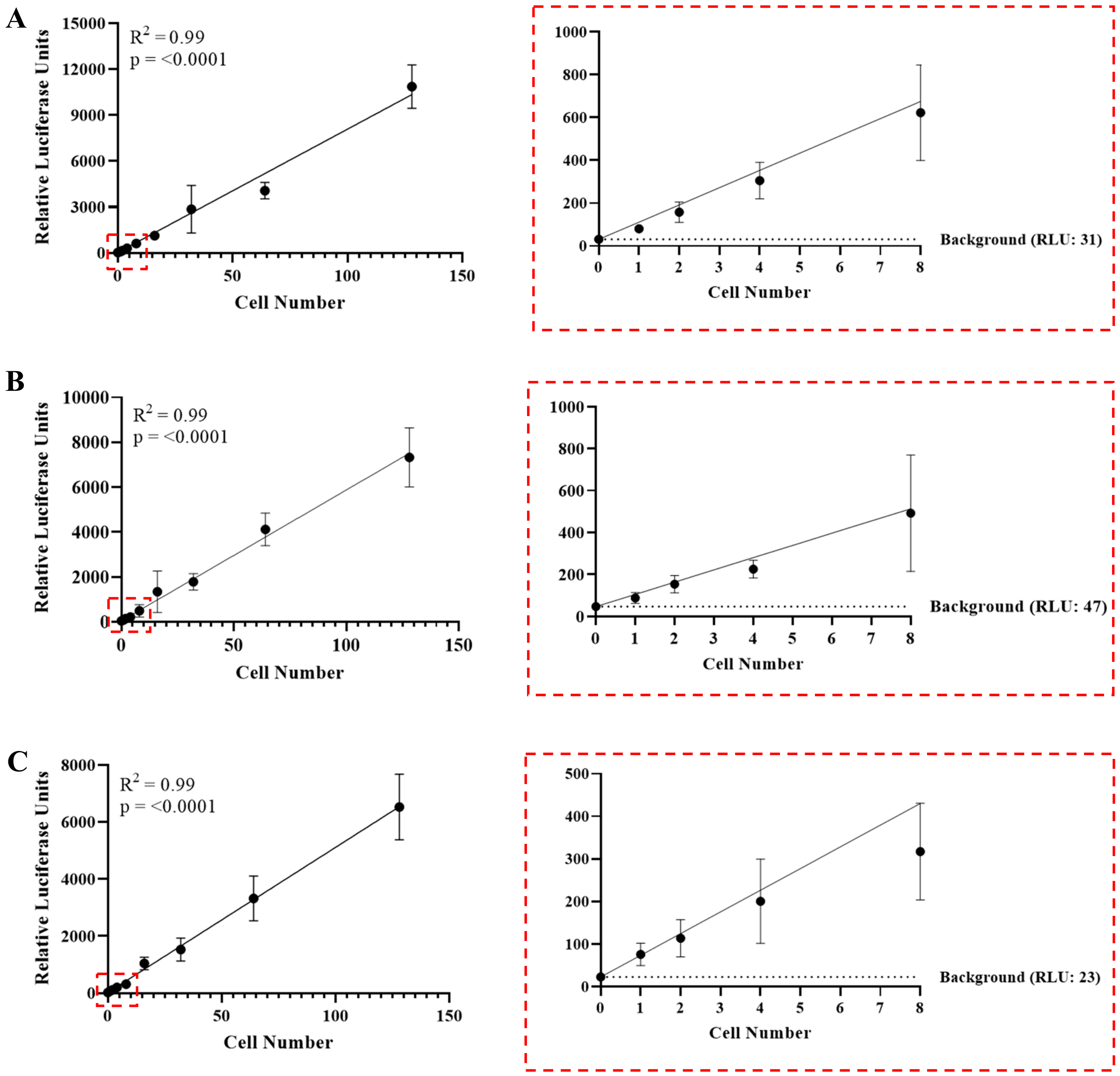

Figure S4: Luciferase-labelled tumour cells spiked in mammary fat pad samples are detected at single-cell level. Detection and quantification of MDA.Gluc **(A),** MCF7.Gluc **(B),** and 4T1.Fluc **(C)** cells spiked in 5 mg of mouse mammary fat pad samples. Left panels show results obtained with all dilution points analysed. Right panels show enlarged regions of left panels, highlighting results obtained with 0-8 cells. Data is represented as mean ± SEM and corresponds to relative luciferase units (RLU) obtained from six readings. Linear correlation was determined by best-fit linear regression analysis.
